# 647. Comparative Analysis of Two Sample -to - Answer Nucleic Acid Amplification Assays for the Detection of Respiratory Viral Pathogens from Pediatric Respiratory Specimens

**DOI:** 10.1093/ofid/ofad500.710

**Published:** 2023-11-27

**Authors:** Anjana Sasidharan, Dithi Banerjee, Amanda M Hayes, Sydnie Petty, Jennifer E Schuster, Jennifer Goldman, Rangaraj Selvarangan

**Affiliations:** Childrens Mercy Hospital, Missouri, Kansas; Children's Mercy Hospital, Kansas City, Missouri; Children's Mercy Hospital, Kansas City, Missouri; MCC-Longview, Raytown, Missouri; Children’s Mercy Kansas City, Kansas City, Missouri; Children's Mercy Hospital, Kansas City, Missouri; Children’s Mercy Kansas City, Kansas City, Missouri

## Abstract

**Background:**

Rapid and accurate diagnosis of acute respiratory infection (ARI) in children is crucial for proper patient management, and nucleic acid amplification tests are the gold standard. Nasal swabs are preferred for surveillance studies due to their ease of use, mainly in children. Our study compares the performance of two sample-to-answer (STA) platforms for detecting multiple viruses in self-collected nasal swabs from symptomatic children.

**Figure:**
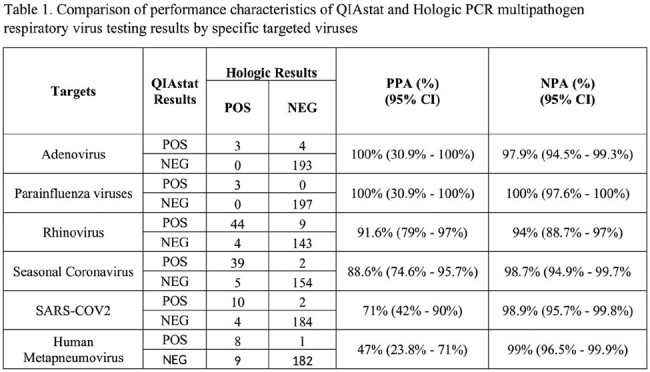

**Methods:**

Between January 2023 and March 2023, self-collected nasal swabs in UTM from children and staff with ARI symptoms were collected as part of an ongoing school-based surveillance study in a large Missouri school district. Samples were tested on the QIAstat-Dx Respiratory SARS-CoV-2 Panel and the fully automated Hologic Panther Fusion system, which can detect a total of 21 respiratory viruses. Percent Agreement analysis was performed to compare the performance of each platform.

**Results:**

Of 200 samples collected, QIAstat detected viruses in 111 samples (55.5%) and Hologic in 115 samples (57.5%). Both assays showed high overall agreement (90%), with a PPA (Positive percentage agreement) of 89%, an NPA (Negative percentage agreement) of 94%. Parainfluenza (PIV) showed 100% PPA and NPA followed by adenovirus (Adv) (PPA: 100%; NPA: 97.9%) and rhinovirus (RV) (PPA: 92% and NPA: 94%). Human metapneumovirus (hMPV) had the lowest PPA (47%) but a high NPA (99%) (Table 1). The median CT values for QIAstat and Hologic were 28.8 (IQR 23.65 - 32.5) and 29.3 (IQR 24.2 – 34.5), respectively. QIAstat missed 19 detections, including hMPV (9, 53%), coronavirus NL63 (4, 16%), and RV (4, 8%), with a corresponding median Ct of 39.1 (IQR 34.5 – 41.2) on Hologic. Hologic missed 12 detections, with RV being the most common target (9, 17%), and a corresponding median Ct of 32.9 (IQR: 30.3-35.5) on QIAstat.

**Conclusion:**

QIAstat and Hologic exhibit comparable performance (overall agreement 90%) for detecting viral respiratory pathogens from self-collected nasal swabs. Viral detections missed by QIAstat assay had low viral loads, while targets missed by Hologic assay had moderate viral load; additional investigation is needed. Our findings suggest that both STA platforms are suitable for diagnosing respiratory viral infections in self-collected nasal swabs in school-setting.

**Disclosures:**

**Rangaraj Selvarangan, BVSc, PhD, D(ABMM), FIDSA, FAAM**, Abbott: Honoraria|Altona Diagnostics: Grant/Research Support|Baebies Inc: Advisor/Consultant|BioMerieux: Advisor/Consultant|BioMerieux: Grant/Research Support|Bio-Rad: Grant/Research Support|Cepheid: Grant/Research Support|GSK: Advisor/Consultant|Hologic: Grant/Research Support|Lab Simply: Advisor/Consultant|Luminex: Grant/Research Support

